# Dichloridobis{*N*,*N*-diethyl-4-[(pyridin-2-yl-κ*N*)diazen­yl]aniline}zinc

**DOI:** 10.1107/S1600536811022884

**Published:** 2011-06-18

**Authors:** Nararak Leesakul, Chaveng Pakawatchai, Saowanit Saithong, Yuthana Tantirungrotechai, Kwanchanok Kwanplod

**Affiliations:** a Department of Chemistry and Center for Innovation in Chemistry, Faculty of Science, Prince of Songkla University, Hat Yai, Songkhla 90112, Thailand; b National Nanotechnology Center, National Science and Technology Development Agency, Thailand Science Park, Klong Luang, Pathumthani 12120, Thailand

## Abstract

In the title complex, [ZnCl_2_(C_15_H_18_N_4_)_2_], the Zn^II^ cation is coordinated by two N atoms from the pyridine rings of two unidentate *N*,*N*-diethyl-4-[(pyridin-2-yl)diazen­yl]aniline ligands and two Cl atoms, resulting in a distorted tetra­hedral geometry. The ligands are mutually transoid with respect to the metal atom. Weak inter­molecular C—H⋯Cl hydrogen bonds and π–π inter­actions, with centroid–centroid distances of 3.8452 (14) and 3.9932 (14) Å, are found in the crystal packing.

## Related literature

For background to azo complexes, see: Arslan (2007 ▶); Santra *et al.* (2001 ▶); Peacock *et al.* (2007 ▶); Ohashi *et al.* (2003 ▶). For applications of azo compounds, see: Millington *et al.* (2007 ▶); Hallas & Choi (1999 ▶); Ho *et al.* (1995 ▶); Sharma *et al.* (2008 ▶). For their photochromic properties, see: Baena *et al.* (1994 ▶). For structures of related azoimine complexes, see: Leesakul *et al.* (2010 ▶); Nag *et al.* (2001 ▶); Pramanik & Das (2010 ▶); Steffen & Palenik (1976 ▶).
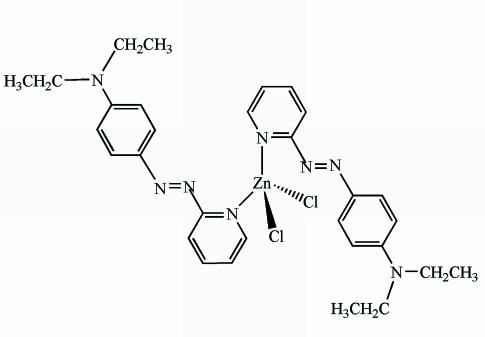


## Experimental

### 

#### Crystal data

[ZnCl_2_(C_15_H_18_N_4_)_2_]
*M* *_r_* = 644.96
Monoclinic, 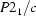

*a* = 13.4058 (6) Å
*b* = 13.8797 (6) Å
*c* = 16.8157 (8) Å
β = 100.562 (1)°
*V* = 3075.9 (2) Å^3^
*Z* = 4
Mo *K*α radiation
μ = 1.01 mm^−1^
*T* = 100 K
0.17 × 0.17 × 0.06 mm

#### Data collection

Bruker APEX CCD area-detector diffractometer
Absorption correction: multi-scan (*SADABS*; Bruker, 2003 ▶) *T*_min_ = 0.780, *T*_max_ = 1.000
32570 measured reflections
5410 independent reflections
4547 reflections with *I* > 2s(*I*)
*R*_int_ = 0.050

#### Refinement

*R*[*F* ^2^ > 2σ(*F* ^2^)] = 0.035
*wR*(*F* ^2^) = 0.086
*S* = 1.06
5410 reflections
374 parameters
H-atom parameters constrained
Δρ_max_ = 0.50 e Å^−3^
Δρ_min_ = −0.27 e Å^−3^

### 

Data collection: *SMART* (Bruker, 1998 ▶); cell refinement: *SAINT* (Bruker, 2003 ▶); data reduction: *SAINT*; program(s) used to solve structure: *SHELXS97* (Sheldrick, 2008 ▶); program(s) used to refine structure: *SHELXL97* (Sheldrick, 2008 ▶); molecular graphics: *Mercury* (Macrae *et al.*, 2008 ▶); software used to prepare material for publication: *SHELXTL* (Sheldrick, 2008 ▶) and *publCIF* (Westrip, 2010 ▶).

## Supplementary Material

Crystal structure: contains datablock(s) I, global. DOI: 10.1107/S1600536811022884/fj2423sup1.cif

Structure factors: contains datablock(s) I. DOI: 10.1107/S1600536811022884/fj2423Isup2.hkl

Additional supplementary materials:  crystallographic information; 3D view; checkCIF report

## Figures and Tables

**Table 1:** Hydrogen-bond geometry (Å, °)

*D*—H⋯*A*	*D*—H	H⋯*A*	*D*⋯*A*	*D*—H⋯*A*
C17—H17⋯Cl1^i^	0.95	2.72	3.486 (2)	138

Symmetry code: (i) 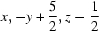.

**Fig. 1.**
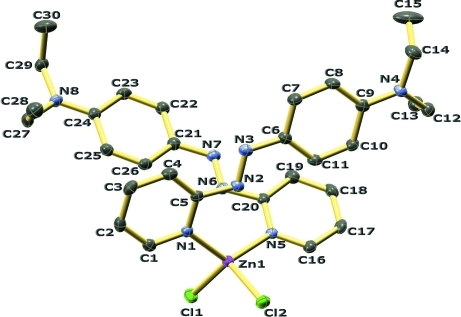
Molecular structure of complex with thermal ellipsoids plotted at the 50% probability level. H atoms are omitted.

**Fig. 2.**
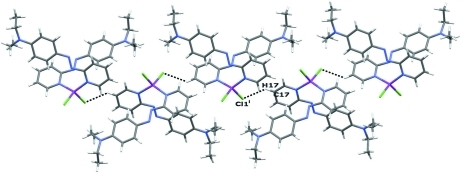
The weak intermolecular interactions of C—H···Cl between the adjacent molecules.

**Fig. 3.**
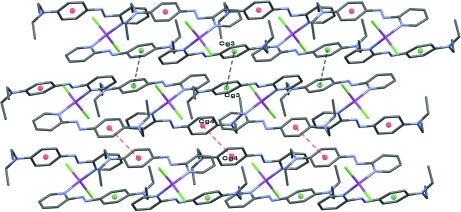
The π–π interactions between molecules in crystal packing.

## supplementary crystallographic information

### Comment

The chemistry of azoimine (—N═N—C═N—) compounds has been known for stabilizing the low-valent metal ions (Arslan, 2007; Santra *et al.*, 2001; Peacock *et al.*,2007; Ohashi *et al.*, 2003). The imine (—C═N—) and azo (—N═N—) units are ordinary high affinity towards transition metal binding *via* N hetero atom. Azo compounds are highly colored and commonly utilized in textile industries (Millington *et al.*, 2007; Hallas *et al.*, 1999), optical data storage (Ho *et al.*, 1995) and sensitizer in DSSC (Sharma *et al.*, 2008). In particular, coordination compounds of Zn^II^ incorporating with azo moiety are extensively used as photoactive materials owing to its interesting photochromic properties (Baena *et al.*, 1994).

Herein, we report the synthesis and crystal structure of a novel Zn^II^ complex with *N*,*N*-diethyl-4-[2-(pyridyl)diazenyl]aniline (C_30_H_36_N_8_: deazpy), an azoimine ligand. The molecular structure of Zn(C_30_H_36_N_8_)Cl_2_ is a distorted tetrahedral complex (Scheme 1 and Fig.1). The *N*,*N*-diethyl-4-[2-(pyridyl)diazenyl]aniline ligand is a bidentate ligand. The chelated coordinations (Nag *et al.*, 2001) between Zn^II^ and N donor atoms of pyridine and azo moieties are generally observed in the crystal structure. However, in the present work, the Zn^II^ coordinates to two unidentate deazpy ligands *via* N(py) atoms [Zn(1)—N(1) = 2.0513 (19) Å, Zn(1)—N(5) = 2.0439 (19) Å] and two Cl atoms [Zn(1)—Cl(1) = 2.2565 (6) Å, Zn(1)—Cl(2) = 2.2713 (6) Å]. These Zn—N bond distances are slightly longer than that of related Zn^II^ with two unidentate imidazole ligands (Pramanik *et al.*, 2010) giving the Zn—N distances = 2.003 (3) and 2.013 (3) Å. The reported Zn—Cl bond distances in dichlorobis(2-azopyridine)zinc(II) (Nag *et al.*, 2001) complex are averaged to 2.2293 Å while the averaged Zn—Cl bond length in complex of dichlorobis(pyridine)zinc(II) reports at 2.222 Å (Steffen *et al.*, 1976) which are slightly shorter than our complex (average 2.2639 Å). All N—Zn—N, N—Zn—Cl and Cl—Zn—Cl bond angles deviate from 109.5¯, especially for N(5)—Zn(1)—N(1) = 123.54 (8)*^o^* arising from the steric constraints from the deazpy structure. The torsion angles of pyridine-azo-phenyl atoms, C(5)—N(2)—N(3)—C(6) and C(20)—N(6)—N(7)—C(21), are -179.03 (19) and -178.30 (19) *^o^*, respectively. The dihedral angle of mean planes of pyridine-azo-phenyl rings among two ligands is 57.40(0.04)*^o^*. Within the ligand molecules, the N(py) atoms exist in *trans*-orientation with respect to the N(azo) atom attached to the phenyl ring. It is as same as that observed from the similar free ligand, *N*,*N*-dimethyl-4-[2(pyridyl)diazenyl] aniline (dmazpy) (Leesakul *et al.*, 2010). The N═N distances of the Zn^II^ complex are 1.286 (3) Å for N2═N3 and 1.280 (3) Å for N6═N7 which are longer than that of the free dmazpy ligand, 1.2566 (16) Å. It is because of the back donation of electron from d^10^-Zn^II^ to π* orbital of the ligands. The strength of the azo bond decreases in comparison with the related free ligand.

The intramolecular C—H···π interactions are found between the phenyl ring of ligand 1 and the pyridine ring (*Cg*2) of ligand 2 [C(11)—H(11)···π*_Cg_*_2_ = 3.303 Å] and *vice versa* [C(26)—H(26)···π*_Cg_*_1_ = 3.550 Å](Fig. 2). In crystal packing, each molecule interacts the adjacent molecules *via* weak hydrogen-bonding interactions of C(17)—H(17)···Cl(1)^i^, [C···Cl = 3.486 (2) Å, symmetry code i: *x*, -*y* + 5/2, *z* - 1/2] (Fig. 2 and Tab. 1).  In addition, the intermolecular π–π interactions are found between the phenyl ring of ligands and the adjacent molecules [Cg3···Cg3^ii^ = 3.8452 (14) Å and Cg4···Cg4^iii^ = 3.9932 (14) Å, symmetry code (ii): 2-*x*, 1-*y*, 1-*z*, (iii): 1-*x*, 1-*y*, -*z*] (Fig. 3 and Tab. 2).

### Experimental

An acetonitrile solution (20 ml) of the *N,N*-diethyl-4-[2-(pyridyl)diazenyl]aniline ligand (0.15 g, 0.6 mmol) and ZnCl_2_ (0.04 g, 0.3 mmol) was refluxed for 4 h. The filtrate was left at room temperature for 2 weeks. The dark red solids were precipitated and washed it with CH_2_Cl_2_ and diethylether, respectively for twice times in order to remove the excess ligands. The dark red solids were recrystallized in acetonitrile and methanol (1:2) at 277 K for 10 days. The red crystals were obtained (yield 67%, 0.13 g).

### Refinement

The structure was solved by direct methods refined by a full-matrix least-squares procedure based on *F^2^*. All hydrogen atoms were constrained, C—H = 0.95 Å with *U*_iso_(H) = 1.2*U*_eq_(C) for C-*sp*^2^ atoms of pyridine and phenyl rings and C—H = 0.98–0.99 Å with *U*_iso_(H) = 1.5*U*_eq_(C) for C-*sp*^3^ atoms of the ethyl group respectively.

### Crystal data

**Table:**

[ZnCl_2_(C_15_H_18_N_4_)_2_]	*F*(000) = 1344
*M**_r_* = 644.96	*D*_x_ = 1.393 Mg m^−^^3^
Monoclinic, *P*2_1_/*c*	Mo *K*α radiation, λ = 0.71073 Å
Hall symbol: -P 2ybc	Cell parameters from 5833 reflections
*a* = 13.4058 (6) Å	θ = 2.3–24.9°
*b* = 13.8797 (6) Å	µ = 1.01 mm^−^^1^
*c* = 16.8157 (8) Å	*T* = 100 K
β = 100.562 (1)°	Block, red brown
*V* = 3075.9 (2) Å^3^	0.17 × 0.17 × 0.06 mm
*Z* = 4	

### Data collection

**Table:**

Bruker APEX CCD area-detector diffractometer	5410 independent reflections
Radiation source: fine-focus sealed tube	4547 reflections with *I* > 2s(*I*)
graphite	*R*_int_ = 0.050
Frames, each covering 0.3 ° in ω scans	θ_max_ = 25.0°, θ_min_ = 1.6°
Absorption correction: multi-scan (*SADABS*; Bruker, 2003)	*h* = −15→15
*T*_min_ = 0.780, *T*_max_ = 1.000	*k* = −16→16
32570 measured reflections	*l* = −19→19

### Refinement

**Table:**

Refinement on *F*^2^	Primary atom site location: structure-invariant direct methods
Least-squares matrix: full	Secondary atom site location: difference Fourier map
*R*[*F*^2^ > 2σ(*F*^2^)] = 0.035	Hydrogen site location: inferred from neighbouring sites
*wR*(*F*^2^) = 0.086	H-atom parameters constrained
*S* = 1.06	*w* = 1/[σ^2^(*F*_o_^2^) + (0.042*P*)^2^ + 1.4983*P*] where *P* = (*F*_o_^2^ + 2*F*_c_^2^)/3
5410 reflections	(Δ/σ)_max_ = 0.002
374 parameters	Δρ_max_ = 0.50 e Å^−^^3^
0 restraints	Δρ_min_ = −0.27 e Å^−^^3^

### Special details

**Table:**

Geometry. All e.s.d.'s (except the e.s.d. in the dihedral angle between two l.s. planes) are estimated using the full covariance matrix. The cell e.s.d.'s are taken into account individually in the estimation of e.s.d.'s in distances, angles and torsion angles; correlations between e.s.d.'s in cell parameters are only used when they are defined by crystal symmetry. An approximate (isotropic) treatment of cell e.s.d.'s is used for estimating e.s.d.'s involving l.s. planes.
Refinement. Refinement of *F*^2^ against all reflections. The weighted *R*-factor *wR* and goodness of fit *S* are based on *F*^2^, conventional *R*-factors *R* are based on *F*, with *F* set to zero for negative *F*^2^. The threshold expression of *F*^2^ > 2*σ* (*F*^2^) is used only for calculating *R*-factors(gt) *etc*. and is not relevant to the choice of reflections for refinement. *R*-factors based on *F*^2^ are statistically about twice as large as those based on *F*, and *R*- factors based on all data will be even larger.

### Fractional atomic coordinates and isotropic or equivalent isotropic displacement parameters (Å^2^)

**Table:**

	*x*	*y*	*z*	*U*_iso_*/*U*_eq_	
Zn1	0.22361 (2)	1.268175 (19)	0.199133 (15)	0.01635 (9)	
Cl1	0.33540 (5)	1.35648 (4)	0.28631 (3)	0.02473 (15)	
Cl2	0.10979 (5)	1.36055 (4)	0.11562 (4)	0.02345 (15)	
N1	0.14219 (14)	1.19205 (13)	0.27007 (11)	0.0166 (4)	
N2	0.11954 (14)	1.08164 (13)	0.16762 (11)	0.0183 (4)	
N3	0.09170 (14)	0.99507 (14)	0.14720 (12)	0.0193 (4)	
N4	0.15013 (15)	0.85544 (14)	−0.15184 (12)	0.0197 (4)	
N5	0.30591 (14)	1.20492 (13)	0.12234 (11)	0.0162 (4)	
N6	0.35399 (14)	1.09944 (13)	0.22628 (11)	0.0175 (4)	
N7	0.40540 (14)	1.02387 (14)	0.25039 (11)	0.0191 (4)	
N8	0.38830 (15)	0.89031 (14)	0.56015 (11)	0.0207 (4)	
C1	0.13247 (17)	1.22457 (17)	0.34345 (14)	0.0191 (5)	
H1	0.1592	1.2862	0.3602	0.023*	
C2	0.08522 (17)	1.17201 (18)	0.39560 (14)	0.0212 (5)	
H2	0.0804	1.1962	0.4476	0.025*	
C3	0.04487 (17)	1.08254 (18)	0.36986 (14)	0.0217 (5)	
H3	0.0132	1.0441	0.4048	0.026*	
C4	0.05098 (17)	1.04982 (17)	0.29372 (14)	0.0204 (5)	
H4	0.0208	0.9903	0.2746	0.025*	
C5	0.10238 (16)	1.10573 (16)	0.24487 (14)	0.0166 (5)	
C6	0.10811 (17)	0.96618 (16)	0.07189 (14)	0.0186 (5)	
C7	0.07776 (18)	0.87198 (17)	0.04831 (15)	0.0219 (5)	
H7	0.0478	0.8329	0.0839	0.026*	
C8	0.09029 (18)	0.83498 (17)	−0.02471 (14)	0.0213 (5)	
H8	0.0678	0.7714	−0.0392	0.026*	
C9	0.13627 (17)	0.89031 (17)	−0.07878 (14)	0.0184 (5)	
C10	0.16746 (18)	0.98555 (17)	−0.05428 (14)	0.0194 (5)	
H10	0.1995	1.0245	−0.0887	0.023*	
C11	0.15197 (17)	1.02167 (17)	0.01785 (14)	0.0196 (5)	
H11	0.1714	1.0862	0.0318	0.024*	
C12	0.21799 (18)	0.90415 (18)	−0.19791 (15)	0.0233 (5)	
H12A	0.1994	0.9732	−0.2032	0.028*	
H12B	0.2079	0.8764	−0.2530	0.028*	
C13	0.32931 (19)	0.89580 (19)	−0.15950 (15)	0.0270 (6)	
H13A	0.3398	0.9221	−0.1045	0.040*	
H13B	0.3704	0.9320	−0.1918	0.040*	
H13C	0.3496	0.8279	−0.1574	0.040*	
C14	0.1117 (2)	0.76118 (18)	−0.18226 (16)	0.0277 (6)	
H14A	0.0902	0.7650	−0.2417	0.033*	
H14B	0.0512	0.7451	−0.1588	0.033*	
C15	0.1883 (2)	0.6822 (2)	−0.1625 (2)	0.0539 (10)	
H15A	0.2444	0.6931	−0.1915	0.081*	
H15B	0.1562	0.6201	−0.1789	0.081*	
H15C	0.2145	0.6816	−0.1041	0.081*	
C16	0.30213 (18)	1.23720 (17)	0.04651 (14)	0.0193 (5)	
H16	0.2644	1.2940	0.0300	0.023*	
C17	0.35077 (17)	1.19130 (17)	−0.00828 (14)	0.0211 (5)	
H17	0.3466	1.2155	−0.0616	0.025*	
C18	0.40618 (18)	1.10855 (18)	0.01682 (14)	0.0221 (5)	
H18	0.4401	1.0751	−0.0197	0.026*	
C19	0.41197 (17)	1.07505 (17)	0.09476 (14)	0.0206 (5)	
H19	0.4504	1.0191	0.1129	0.025*	
C20	0.36016 (17)	1.12516 (16)	0.14622 (13)	0.0167 (5)	
C21	0.39913 (17)	0.99451 (17)	0.32815 (14)	0.0187 (5)	
C22	0.45412 (18)	0.91200 (17)	0.35774 (14)	0.0208 (5)	
H22	0.4943	0.8800	0.3249	0.025*	
C23	0.45074 (17)	0.87670 (17)	0.43350 (14)	0.0207 (5)	
H23	0.4885	0.8205	0.4519	0.025*	
C24	0.39216 (17)	0.92230 (16)	0.48482 (14)	0.0188 (5)	
C25	0.33610 (18)	1.00572 (17)	0.45299 (14)	0.0200 (5)	
H25	0.2948	1.0377	0.4850	0.024*	
C26	0.34047 (18)	1.04044 (17)	0.37802 (14)	0.0194 (5)	
H26	0.3032	1.0967	0.3591	0.023*	
C27	0.31848 (19)	0.93302 (18)	0.60798 (14)	0.0245 (6)	
H27A	0.3236	1.0041	0.6054	0.029*	
H27B	0.3399	0.9136	0.6652	0.029*	
C28	0.20876 (19)	0.9040 (2)	0.57998 (16)	0.0312 (6)	
H28A	0.1865	0.9235	0.5235	0.047*	
H28B	0.1664	0.9357	0.6139	0.047*	
H28C	0.2024	0.8339	0.5844	0.047*	
C29	0.44691 (19)	0.80678 (17)	0.59611 (15)	0.0239 (6)	
H29A	0.4663	0.8172	0.6552	0.029*	
H29B	0.5102	0.8027	0.5738	0.029*	
C30	0.3912 (2)	0.71195 (18)	0.5815 (2)	0.0381 (7)	
H30A	0.3286	0.7150	0.6037	0.057*	
H30B	0.4344	0.6600	0.6081	0.057*	
H30C	0.3744	0.6994	0.5232	0.057*	

### Atomic displacement parameters (Å^2^)

**Table:**

	*U*^11^	*U*^22^	*U*^33^	*U*^12^	*U*^13^	*U*^23^
Zn1	0.01989 (16)	0.01422 (15)	0.01534 (15)	0.00023 (11)	0.00428 (11)	0.00035 (11)
Cl1	0.0290 (3)	0.0241 (3)	0.0210 (3)	−0.0076 (3)	0.0041 (3)	−0.0036 (2)
Cl2	0.0261 (3)	0.0200 (3)	0.0243 (3)	0.0059 (2)	0.0047 (3)	0.0049 (2)
N1	0.0165 (10)	0.0162 (10)	0.0170 (10)	0.0029 (8)	0.0026 (8)	0.0020 (8)
N2	0.0170 (10)	0.0169 (10)	0.0204 (10)	0.0005 (8)	0.0013 (8)	−0.0017 (8)
N3	0.0181 (10)	0.0159 (10)	0.0230 (11)	0.0003 (8)	0.0017 (8)	−0.0014 (8)
N4	0.0200 (10)	0.0182 (10)	0.0205 (10)	−0.0021 (8)	0.0024 (8)	−0.0033 (8)
N5	0.0169 (10)	0.0143 (10)	0.0173 (10)	−0.0024 (8)	0.0029 (8)	−0.0008 (8)
N6	0.0170 (10)	0.0163 (10)	0.0187 (10)	−0.0011 (8)	0.0021 (8)	0.0009 (8)
N7	0.0180 (10)	0.0170 (10)	0.0215 (10)	0.0008 (8)	0.0010 (8)	0.0001 (8)
N8	0.0229 (11)	0.0183 (10)	0.0208 (11)	0.0040 (9)	0.0040 (9)	0.0037 (9)
C1	0.0162 (12)	0.0191 (12)	0.0210 (13)	0.0032 (10)	0.0011 (10)	−0.0005 (10)
C2	0.0180 (12)	0.0288 (14)	0.0171 (12)	0.0060 (11)	0.0042 (10)	0.0015 (10)
C3	0.0156 (12)	0.0256 (13)	0.0247 (13)	0.0022 (10)	0.0059 (10)	0.0085 (11)
C4	0.0139 (12)	0.0167 (12)	0.0299 (14)	−0.0002 (10)	0.0022 (10)	0.0014 (10)
C5	0.0133 (12)	0.0150 (12)	0.0202 (12)	0.0015 (9)	−0.0001 (9)	0.0022 (10)
C6	0.0160 (12)	0.0177 (12)	0.0213 (12)	0.0006 (10)	0.0017 (10)	−0.0017 (10)
C7	0.0224 (13)	0.0177 (12)	0.0259 (13)	−0.0030 (10)	0.0056 (10)	0.0014 (10)
C8	0.0221 (13)	0.0141 (12)	0.0277 (13)	−0.0020 (10)	0.0047 (10)	−0.0021 (10)
C9	0.0123 (11)	0.0186 (12)	0.0224 (12)	0.0019 (10)	−0.0015 (9)	0.0000 (10)
C10	0.0191 (12)	0.0189 (12)	0.0196 (12)	−0.0035 (10)	0.0022 (10)	0.0022 (10)
C11	0.0172 (12)	0.0141 (12)	0.0260 (13)	−0.0015 (10)	0.0001 (10)	−0.0005 (10)
C12	0.0248 (14)	0.0237 (13)	0.0210 (13)	−0.0022 (11)	0.0035 (10)	−0.0018 (10)
C13	0.0260 (14)	0.0278 (14)	0.0279 (14)	−0.0030 (11)	0.0072 (11)	−0.0005 (11)
C14	0.0265 (14)	0.0237 (14)	0.0326 (15)	−0.0047 (11)	0.0049 (11)	−0.0080 (11)
C15	0.0332 (17)	0.0193 (15)	0.103 (3)	−0.0011 (13)	−0.0038 (18)	−0.0061 (17)
C16	0.0194 (12)	0.0182 (12)	0.0203 (12)	−0.0038 (10)	0.0033 (10)	0.0014 (10)
C17	0.0208 (13)	0.0247 (13)	0.0182 (12)	−0.0094 (11)	0.0048 (10)	0.0022 (10)
C18	0.0204 (13)	0.0236 (13)	0.0241 (13)	−0.0039 (11)	0.0087 (10)	−0.0074 (11)
C19	0.0166 (12)	0.0204 (12)	0.0256 (13)	−0.0011 (10)	0.0056 (10)	−0.0022 (10)
C20	0.0159 (12)	0.0149 (12)	0.0193 (12)	−0.0042 (10)	0.0031 (9)	−0.0009 (9)
C21	0.0165 (12)	0.0186 (12)	0.0204 (12)	−0.0004 (10)	0.0015 (10)	0.0000 (10)
C22	0.0184 (12)	0.0207 (13)	0.0239 (13)	0.0024 (10)	0.0055 (10)	−0.0005 (10)
C23	0.0176 (12)	0.0171 (12)	0.0265 (13)	0.0051 (10)	0.0020 (10)	0.0022 (10)
C24	0.0186 (12)	0.0177 (12)	0.0187 (12)	−0.0010 (10)	−0.0002 (10)	−0.0002 (10)
C25	0.0203 (12)	0.0183 (12)	0.0214 (12)	0.0026 (10)	0.0036 (10)	−0.0022 (10)
C26	0.0196 (13)	0.0153 (12)	0.0221 (12)	0.0024 (10)	0.0005 (10)	0.0014 (10)
C27	0.0313 (14)	0.0255 (13)	0.0175 (12)	0.0064 (11)	0.0068 (11)	0.0009 (10)
C28	0.0270 (15)	0.0318 (15)	0.0367 (16)	0.0058 (12)	0.0109 (12)	0.0037 (12)
C29	0.0267 (14)	0.0232 (13)	0.0211 (13)	0.0051 (11)	0.0026 (10)	0.0043 (10)
C30	0.0363 (17)	0.0194 (14)	0.058 (2)	0.0035 (12)	0.0057 (14)	0.0040 (13)

### Geometric parameters (Å, °)

**Table:**

Zn1—N5	2.0439 (19)	C12—H12B	0.9900
Zn1—N1	2.0513 (19)	C13—H13A	0.9800
Zn1—Cl1	2.2565 (6)	C13—H13B	0.9800
Zn1—Cl2	2.2713 (6)	C13—H13C	0.9800
N1—C1	1.343 (3)	C14—C15	1.498 (4)
N1—C5	1.348 (3)	C14—H14A	0.9900
N2—N3	1.286 (3)	C14—H14B	0.9900
N2—C5	1.401 (3)	C15—H15A	0.9800
N3—C6	1.384 (3)	C15—H15B	0.9800
N4—C9	1.364 (3)	C15—H15C	0.9800
N4—C14	1.463 (3)	C16—C17	1.378 (3)
N4—C12	1.464 (3)	C16—H16	0.9500
N5—C16	1.344 (3)	C17—C18	1.391 (3)
N5—C20	1.345 (3)	C17—H17	0.9500
N6—N7	1.280 (3)	C18—C19	1.379 (3)
N6—C20	1.410 (3)	C18—H18	0.9500
N7—C21	1.387 (3)	C19—C20	1.391 (3)
N8—C24	1.352 (3)	C19—H19	0.9500
N8—C27	1.466 (3)	C21—C22	1.403 (3)
N8—C29	1.467 (3)	C21—C26	1.404 (3)
C1—C2	1.381 (3)	C22—C23	1.373 (3)
C1—H1	0.9500	C22—H22	0.9500
C2—C3	1.391 (3)	C23—C24	1.418 (3)
C2—H2	0.9500	C23—H23	0.9500
C3—C4	1.375 (3)	C24—C25	1.430 (3)
C3—H3	0.9500	C25—C26	1.361 (3)
C4—C5	1.400 (3)	C25—H25	0.9500
C4—H4	0.9500	C26—H26	0.9500
C6—C11	1.400 (3)	C27—C28	1.514 (4)
C6—C7	1.405 (3)	C27—H27A	0.9900
C7—C8	1.369 (3)	C27—H27B	0.9900
C7—H7	0.9500	C28—H28A	0.9800
C8—C9	1.415 (3)	C28—H28B	0.9800
C8—H8	0.9500	C28—H28C	0.9800
C9—C10	1.424 (3)	C29—C30	1.511 (4)
C10—C11	1.363 (3)	C29—H29A	0.9900
C10—H10	0.9500	C29—H29B	0.9900
C11—H11	0.9500	C30—H30A	0.9800
C12—C13	1.518 (3)	C30—H30B	0.9800
C12—H12A	0.9900	C30—H30C	0.9800
			
N5—Zn1—N1	123.54 (8)	N4—C14—H14A	108.9
N5—Zn1—Cl1	105.82 (5)	C15—C14—H14A	108.9
N1—Zn1—Cl1	105.24 (6)	N4—C14—H14B	108.9
N5—Zn1—Cl2	103.36 (6)	C15—C14—H14B	108.9
N1—Zn1—Cl2	106.35 (5)	H14A—C14—H14B	107.8
Cl1—Zn1—Cl2	112.70 (2)	C14—C15—H15A	109.5
C1—N1—C5	119.2 (2)	C14—C15—H15B	109.5
C1—N1—Zn1	120.86 (16)	H15A—C15—H15B	109.5
C5—N1—Zn1	119.82 (15)	C14—C15—H15C	109.5
N3—N2—C5	112.43 (19)	H15A—C15—H15C	109.5
N2—N3—C6	115.34 (19)	H15B—C15—H15C	109.5
C9—N4—C14	122.3 (2)	N5—C16—C17	122.7 (2)
C9—N4—C12	120.86 (19)	N5—C16—H16	118.7
C14—N4—C12	116.16 (19)	C17—C16—H16	118.7
C16—N5—C20	118.8 (2)	C16—C17—C18	118.1 (2)
C16—N5—Zn1	121.70 (16)	C16—C17—H17	121.0
C20—N5—Zn1	119.34 (15)	C18—C17—H17	121.0
N7—N6—C20	112.80 (18)	C19—C18—C17	120.0 (2)
N6—N7—C21	114.67 (19)	C19—C18—H18	120.0
C24—N8—C27	121.26 (19)	C17—C18—H18	120.0
C24—N8—C29	122.4 (2)	C18—C19—C20	118.4 (2)
C27—N8—C29	116.16 (19)	C18—C19—H19	120.8
N1—C1—C2	122.6 (2)	C20—C19—H19	120.8
N1—C1—H1	118.7	N5—C20—C19	122.0 (2)
C2—C1—H1	118.7	N5—C20—N6	111.74 (19)
C1—C2—C3	118.1 (2)	C19—C20—N6	126.2 (2)
C1—C2—H2	120.9	N7—C21—C22	117.1 (2)
C3—C2—H2	120.9	N7—C21—C26	124.6 (2)
C4—C3—C2	119.9 (2)	C22—C21—C26	118.3 (2)
C4—C3—H3	120.1	C23—C22—C21	121.0 (2)
C2—C3—H3	120.1	C23—C22—H22	119.5
C3—C4—C5	118.9 (2)	C21—C22—H22	119.5
C3—C4—H4	120.6	C22—C23—C24	121.4 (2)
C5—C4—H4	120.6	C22—C23—H23	119.3
N1—C5—C4	121.2 (2)	C24—C23—H23	119.3
N1—C5—N2	112.38 (19)	N8—C24—C23	123.0 (2)
C4—C5—N2	126.5 (2)	N8—C24—C25	120.6 (2)
N3—C6—C11	126.2 (2)	C23—C24—C25	116.4 (2)
N3—C6—C7	116.2 (2)	C26—C25—C24	121.6 (2)
C11—C6—C7	117.6 (2)	C26—C25—H25	119.2
C8—C7—C6	121.7 (2)	C24—C25—H25	119.2
C8—C7—H7	119.2	C25—C26—C21	121.1 (2)
C6—C7—H7	119.2	C25—C26—H26	119.4
C7—C8—C9	120.8 (2)	C21—C26—H26	119.4
C7—C8—H8	119.6	N8—C27—C28	113.8 (2)
C9—C8—H8	119.6	N8—C27—H27A	108.8
N4—C9—C8	122.2 (2)	C28—C27—H27A	108.8
N4—C9—C10	120.6 (2)	N8—C27—H27B	108.8
C8—C9—C10	117.2 (2)	C28—C27—H27B	108.8
C11—C10—C9	120.9 (2)	H27A—C27—H27B	107.7
C11—C10—H10	119.5	C27—C28—H28A	109.5
C9—C10—H10	119.5	C27—C28—H28B	109.5
C10—C11—C6	121.7 (2)	H28A—C28—H28B	109.5
C10—C11—H11	119.1	C27—C28—H28C	109.5
C6—C11—H11	119.1	H28A—C28—H28C	109.5
N4—C12—C13	113.4 (2)	H28B—C28—H28C	109.5
N4—C12—H12A	108.9	N8—C29—C30	114.2 (2)
C13—C12—H12A	108.9	N8—C29—H29A	108.7
N4—C12—H12B	108.9	C30—C29—H29A	108.7
C13—C12—H12B	108.9	N8—C29—H29B	108.7
H12A—C12—H12B	107.7	C30—C29—H29B	108.7
C12—C13—H13A	109.5	H29A—C29—H29B	107.6
C12—C13—H13B	109.5	C29—C30—H30A	109.5
H13A—C13—H13B	109.5	C29—C30—H30B	109.5
C12—C13—H13C	109.5	H30A—C30—H30B	109.5
H13A—C13—H13C	109.5	C29—C30—H30C	109.5
H13B—C13—H13C	109.5	H30A—C30—H30C	109.5
N4—C14—C15	113.2 (2)	H30B—C30—H30C	109.5
			
N5—Zn1—N1—C1	146.45 (16)	C9—C10—C11—C6	2.1 (4)
Cl1—Zn1—N1—C1	25.15 (18)	N3—C6—C11—C10	178.1 (2)
Cl2—Zn1—N1—C1	−94.64 (17)	C7—C6—C11—C10	−1.7 (3)
N5—Zn1—N1—C5	−29.95 (19)	C9—N4—C12—C13	69.6 (3)
Cl1—Zn1—N1—C5	−151.25 (15)	C14—N4—C12—C13	−101.3 (2)
Cl2—Zn1—N1—C5	88.96 (16)	C9—N4—C14—C15	−93.7 (3)
C5—N2—N3—C6	−179.03 (19)	C12—N4—C14—C15	77.0 (3)
N1—Zn1—N5—C16	135.03 (17)	C20—N5—C16—C17	0.7 (3)
Cl1—Zn1—N5—C16	−103.94 (17)	Zn1—N5—C16—C17	−174.90 (17)
Cl2—Zn1—N5—C16	14.72 (18)	N5—C16—C17—C18	−0.3 (3)
N1—Zn1—N5—C20	−40.56 (19)	C16—C17—C18—C19	−0.5 (3)
Cl1—Zn1—N5—C20	80.47 (16)	C17—C18—C19—C20	0.9 (3)
Cl2—Zn1—N5—C20	−160.87 (15)	C16—N5—C20—C19	−0.3 (3)
C20—N6—N7—C21	−178.30 (19)	Zn1—N5—C20—C19	175.45 (17)
C5—N1—C1—C2	1.6 (3)	C16—N5—C20—N6	−178.30 (19)
Zn1—N1—C1—C2	−174.77 (17)	Zn1—N5—C20—N6	−2.6 (2)
N1—C1—C2—C3	−1.1 (3)	C18—C19—C20—N5	−0.5 (3)
C1—C2—C3—C4	−1.3 (3)	C18—C19—C20—N6	177.2 (2)
C2—C3—C4—C5	3.0 (3)	N7—N6—C20—N5	−179.76 (18)
C1—N1—C5—C4	0.2 (3)	N7—N6—C20—C19	2.3 (3)
Zn1—N1—C5—C4	176.66 (16)	N6—N7—C21—C22	−179.9 (2)
C1—N1—C5—N2	−179.48 (19)	N6—N7—C21—C26	1.3 (3)
Zn1—N1—C5—N2	−3.0 (2)	N7—C21—C22—C23	−178.7 (2)
C3—C4—C5—N1	−2.5 (3)	C26—C21—C22—C23	0.1 (3)
C3—C4—C5—N2	177.1 (2)	C21—C22—C23—C24	−0.3 (4)
N3—N2—C5—N1	172.75 (18)	C27—N8—C24—C23	−173.3 (2)
N3—N2—C5—C4	−6.9 (3)	C29—N8—C24—C23	1.7 (3)
N2—N3—C6—C11	0.5 (3)	C27—N8—C24—C25	6.9 (3)
N2—N3—C6—C7	−179.7 (2)	C29—N8—C24—C25	−178.1 (2)
N3—C6—C7—C8	−179.8 (2)	C22—C23—C24—N8	−179.0 (2)
C11—C6—C7—C8	0.0 (4)	C22—C23—C24—C25	0.8 (3)
C6—C7—C8—C9	1.1 (4)	N8—C24—C25—C26	178.6 (2)
C14—N4—C9—C8	4.7 (3)	C23—C24—C25—C26	−1.3 (3)
C12—N4—C9—C8	−165.6 (2)	C24—C25—C26—C21	1.1 (4)
C14—N4—C9—C10	−174.8 (2)	N7—C21—C26—C25	178.2 (2)
C12—N4—C9—C10	14.9 (3)	C22—C21—C26—C25	−0.5 (3)
C7—C8—C9—N4	179.8 (2)	C24—N8—C27—C28	74.3 (3)
C7—C8—C9—C10	−0.7 (3)	C29—N8—C27—C28	−101.0 (2)
N4—C9—C10—C11	178.6 (2)	C24—N8—C29—C30	−91.3 (3)
C8—C9—C10—C11	−0.9 (3)	C27—N8—C29—C30	84.0 (3)

### Hydrogen-bond geometry (Å, °)

**Table:**

*D*—H···*A*	*D*—H	H···*A*	*D*···*A*	*D*—H···*A*
C17—H17···Cl1^i^	0.95	2.72	3.486 (2)	138

Symmetry codes: (i) *x*, −*y*+5/2, *z*−1/2.
